# The Effects of Herbarium Specimen Characteristics on Short-Read NGS Sequencing Success in Nearly 8000 Specimens: Old, Degraded Samples Have Lower DNA Yields but Consistent Sequencing Success

**DOI:** 10.3389/fpls.2021.669064

**Published:** 2021-06-23

**Authors:** Heather R. Kates, Joshua R. Doby, Carol M. Siniscalchi, Raphael LaFrance, Douglas E. Soltis, Pamela S. Soltis, Robert P. Guralnick, Ryan A. Folk

**Affiliations:** ^1^Florida Museum of Natural History, University of Florida, Gainesville, FL, United States; ^2^Department of Biological Sciences, Mississippi State University, Mississippi State, MS, United States; ^3^Department of Biology, University of Florida, Gainesville, FL, United States; ^4^Genetics Institute, University of Florida, Gainesville, FL, United States; ^5^Biodiversity Institute, University of Florida, Gainesville, FL, United States

**Keywords:** herbarium specimens, historical collections, target capture, hyb-seq, DNA yield, global-scale phylogenetics

## Abstract

Phylogenetic datasets are now commonly generated using short-read sequencing technologies unhampered by degraded DNA, such as that often extracted from herbarium specimens. The compatibility of these methods with herbarium specimens has precipitated an increase in broad sampling of herbarium specimens for inclusion in phylogenetic studies. Understanding which sample characteristics are predictive of sequencing success can guide researchers in the selection of tissues and specimens most likely to yield good results. Multiple recent studies have considered the relationship between sample characteristics and DNA yield and sequence capture success. Here we report an analysis of the relationship between sample characteristics and sequencing success for nearly 8,000 herbarium specimens. This study, the largest of its kind, is also the first to include a measure of specimen quality (“greenness”) as a predictor of DNA sequencing success. We found that taxonomic group and source herbarium are strong predictors of both DNA yield and sequencing success and that the most important specimen characteristics for predicting success differ for DNA yield and sequencing: greenness was the strongest predictor of DNA yield, and age was the strongest predictor of proportion-on-target reads recovered. Surprisingly, the relationship between age and proportion-on-target reads is the inverse of expectations; older specimens performed slightly better in our capture-based protocols. We also found that DNA yield itself is not a strong predictor of sequencing success. Most literature on DNA sequencing from herbarium specimens considers specimen selection for optimal DNA extraction success, which we find to be an inappropriate metric for predicting success using next-generation sequencing technologies.

## Introduction

Herbarium specimens and short-read Next-Generation Sequencing (NGS) are natural partners. Phylogenetic projects at the largest scales often cannot be reasonably assembled through field collections. Herbaria provide a way to overcome such limitations because they contain centuries of accumulated genetic material that covers vast stretches of space and time. However, because previous-generation sequencing approaches, such as Sanger sequencing, could not be easily optimized for degraded material (Staats et al., 2013; Jones and Good, 2016), the utility of herbaria for large-scale phylogenetics studies was limited. The burgeoning spectrum of NGS methods has dramatically lowered technical barriers to sequencing degraded materials. Short-read NGS methods are compatible with low input DNA quantities and fragmented DNA molecules that may result from specimen degradation over time (Pyle and Adams, 1989; Savolainen et al., 1995). The broader adoption of short-read NGS methods by much of the phylogenetics community repositions herbarium collections as the primary source for generation of low-copy nuclear DNA datasets (Hart et al., 2016; Zeng et al., 2018; Brewer et al., 2019), enabling comprehensive and global-scale plant phylogenies and other molecular applications (e.g., Brewer et al., 2019; Folk et al., 2021).

Despite this enthusiasm for the use of herbarium resources, we still lack a consensus on the best predictors of herbarium specimen success in DNA isolation and sequencing applications that is needed to broadly inform project design and curatorial practice in a museum setting. Specimen characteristics frequently identified as predictive of DNA extraction and sequencing success include sample age, specimen preservation method, storage climate, genome size, and physical traits specific to a taxonomic group (e.g., leaf texture or presence of secondary compounds; Staats et al., 2011; Neubig et al., 2014; Bakker et al., 2016; Hart et al., 2016; Kuzmina et al., 2017; Forrest et al., 2019). While these predictors have been discussed in previous literature, there are few quantitative studies considering multiple predictors simultaneously, and much of the literature pertains to Sanger sequencing, which requires a high quantity of input DNA and suitably long DNA fragments.

In an era in which herbarium specimens are increasingly sampled for downstream NGS applications, most commonly targeted-enrichment sequencing or “Hyb-Seq,” it is necessary to distinguish between factors that impact DNA extraction yield from those that are related to enrichment and sequencing success, as we should not necessarily expect concordance across these factors. For example, the presence of secondary compounds may not impact DNA yield but may inhibit hybridization assays (Brewer et al., 2019). Small amounts of degraded tissue may yield low amounts of DNA from DNA extraction protocols, but these same characteristics are not guaranteed to be predictive of sequencing success; neither high-molecular-weight DNA nor large quantities of input DNA are necessarily requirements for targeted sequencing applications using short-read sequencing technologies (Rykalina et al., 2014; Song et al., 2018).

Among factors that impact DNA yield, greenness of herbarium specimens is typically considered a valuable predictor of high yield and limited degradation. Leaf greenness is thought to be suggestive of quality as it may indicate the method and speed of drying (Jankowiak et al., 2005); brown specimens often indicate drying under high heat or after ethanol treatment, potentially yielding degraded DNA samples (Erkens et al., 2008). Despite the routine qualitative assessment of herbarium samples by researchers and curators for destructive sampling decisions, efforts to identify specimen characteristics that are predictive of DNA extraction and sequencing success most often consider age rather than greenness even though studies have shown that the method of drying (as indicated by the color of the leaves) was more important for isolation of DNA than the age of the sample (Jankowiak et al., 2005).

As part of a large-scale phylogenomic effort focused on the nitrogen-fixing clade of angiosperms (the clade comprising Cucurbitales, Fabales, Fagales, and Rosales within the rosids (APG IV: Chase et al., 2016), we have collected leaf tissue and sequenced DNA from thousands of herbarium specimens. With broad taxonomic coverage across a clade of > 30,000 species and specimens collected over more than a century across the globe, this effort is an excellent opportunity to assess factors that best predict the success of a standard Hyb-Seq experiment. Collating a dataset comprising localities, specimen photographs, and sequencing QC for thousands of species, we use a mixed modeling framework to test several predictors thought to be important for sequencing success: taxonomic family (following APG IV: Chase et al., 2016), specimen greenness, latitude of the collected specimen, and specimen age. We also included source herbarium as an effect to consider whether curatorial practices or the scope of collections housed may be associated with sequencing success. We test the association of these factors with two commonly used QC metrics used in Hyb-Seq: DNA yield and proportion-on-target reads recovered. We present a series of recommendations with these results with the aim of informing both successful project design and curatorial practices that maximize the usefulness of scientific collections.

## Materials and Methods

### Specimen Sampling

**FIGURE 1:**
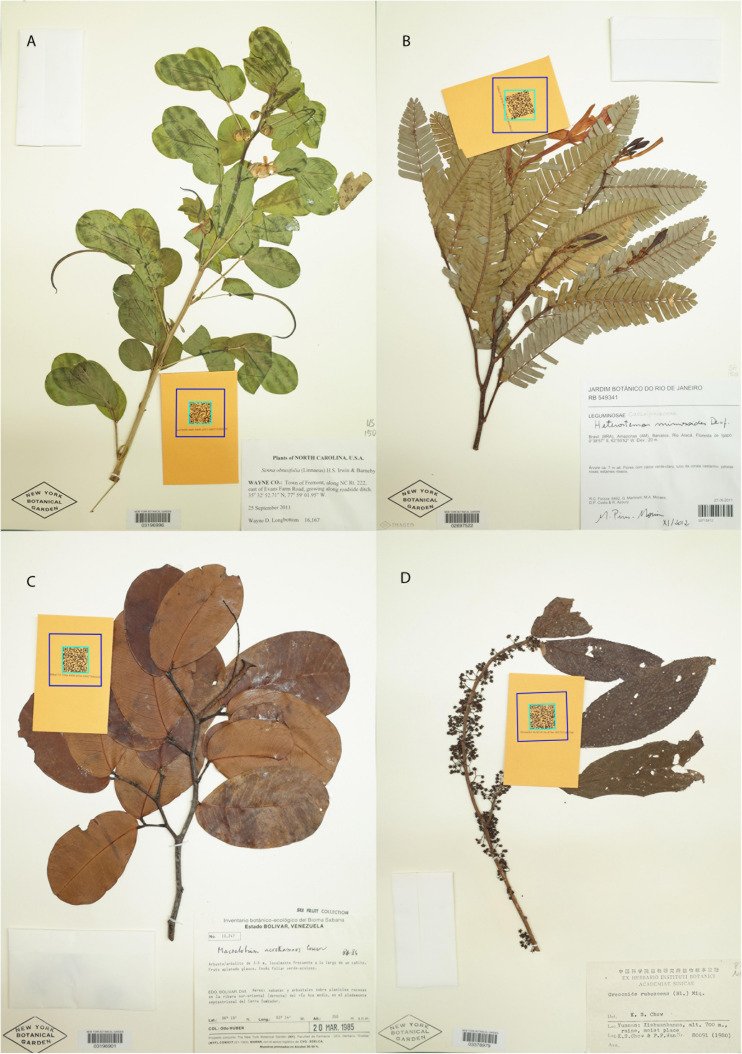
Examples of the four most common color bins: nice green **(A)**, faded green **(B)**, brown **(C)**, and brown-black **(D)**. **(A–D)** Are all three-way consensus scores and therefore are well representative of their respective bins.

The dataset we present comprises 7,608 specimens that are a random subset of the 15,000 herbarium specimens collected as part of the Nitfix project (see Folk et al., 2021), a study that includes ∼50% of the species diversity of the nitrogen-fixing clade. The specimens included here are those for which we have the complete specimen data necessary to fit models that include sampling location and date of collection. Teams of 2–5 researchers sampled specimens from six herbaria in the United States (NY, FLAS, CAS, GH, MO, and OS). Whenever possible, we selected the greenest specimen available for a target taxon, but for many taxa, green specimens were unavailable, and thus our dataset includes a broad spread from bright green to highly discolored brown or black specimens (Figure 1 and Supplementary Figure 1). Specimens were stored in coin envelopes at room temperature in the dark for 1 week to 1 year prior to DNA extraction and sequencing. For a detailed description of the taxonomic sampling and specimen sampling workflow, see Folk et al. (2021).

### Specimen Metadata Generation

We derived specimen age and location from metadata recorded from specimen photographs in a citizen science approach on the Notes from Nature platform (Hill et al., 2012). For a detailed description of the label transcription workflow, see Folk et al. (2021). We converted collection data to years before present via functions in the lubridate R package (Grolemund and Wickham, 2011). We also assembled a latitude and longitude estimate for all specimens lacking an existing georeference via geocoding using the ggmap function in R (Kahle et al., 2019). In particular, we used the lowest administrative unit recorded (ordered from county to state to country) to generate a centroid latitude and longitude for that unit, yielding coarse georeferences sufficient for our global scope of analysis. We checked for issues with geocoding once completed and removed any obvious failures. Finally, we took the absolute value of latitude and used this as a metric for distance from the Equator. We included the following predictors of DNA yield and sequencing success in our analysis: anonymized herbarium (“Herbarium”), age of specimen in years before present (“Specimen.Age”), and distance from Equator (“Absolute.Latitude”).

### Scoring Specimen Greenness

In addition to the voucher metadata considered as predictors of DNA yield and sequencing success, we also recorded a greenness score (“Greenness”) for all specimens included in this study. Because herbarium images were taken in different lighting conditions and the specimens themselves are of different ages, we normalized the color before recording greenness. We did not have access to color chips; we therefore used the manila sampling envelopes that were included in the photos as a color guide to standardize color balance across all specimens. Color scores were limited to five color bins (1:“nice green,” 2:“faded green,” 3:“brown,” 4:“brown-black,” and 5:“black”; Supplementary Figure 1). We trained volunteers to use this scoring rubric and tested volunteers after training sessions to ensure scores were reproducible among different individuals. To account for the remaining variability among scorers, three people were asked to score each set of herbarium specimens; these scores were assigned a numerical value, and the value of “Greenness” was taken as the average across the three scorers. Volunteers scored images using the program imageAnt (Stucky et al., 2019), where each herbarium specimen is assigned a color bin, marked as uncertain, or flagged for discussion. Specimens with any score of “uncertain” or “flagged for discussion” were removed from the dataset. We used the gsubfn function in tidyr (Wickham, 2020) along with core R functions to convert greenness bins reported as text into the averaged numerical scores used in this analysis; “Greenness” scores were ordered from the brightest green specimens (“nice green” = 1.0) to most discolored (“black” = 5.0).

### DNA Extraction, Library Building, Targeted Enrichment, and Sequencing

DNA extraction was performed in a 96-well format using the Genesee Scientific (Rochester, New York, United States) 1.2 mL mini tube system with two 4-mm stainless steel grinding beads per tube. We ground 20–30 mg of herbarium tissue into a fine powder using a MiniG Automated Tissue Homogenizer (SPEX SamplePrep, Metuchen, New Jersey, United States) at 1,500 rpm for 60–120 s at room temperature. Thorough homogenization of dry herbarium specimen tissue is critical for adequate penetration of extraction reagents to enable isolation of sufficient quantities of DNA for NGS. Following grinding, 500 μL of 2 × cetyltrimethylammonium bromide (CTAB) buffer was added to each tube using a Rainin E4 12-channel pipette (Mettler Toledo, Columbus, Ohio, United States). Samples were again homogenized at 900 rpm for 20 s and incubated at 65°C for 60 min in an incubation oven. After incubation, the lysate was transferred to a new set of tubes to reduce the total volume and allow for the addition of chloroform without tube overflow. DNA was isolated and purified twice by adding an equal volume of 24:1 chloroform:isoamyl alcohol and transferring supernatant to a new set of tubes. DNA was precipitated by 8–24 h incubation at -20°C with 0.08 volume of cold 7.5 M ammonium acetate and 0.54 volume of cold isopropanol. DNA pellets were washed two times with 500 μL cold 70% ethanol, and dried DNA pellets were resuspended in 33 μL of molecular grade water. Extracts were transferred immediately to 96-well microplates using an LTS 12-channel pipette, and plates were sealed with alumaseal foil.

Samples were briefly stored at -20°C and submitted to RAPiD Genomics (Gainesville, Florida, United States) for quantification, library preparation, targeted enrichment using a custom biotinylated RNA bait set (Folk et al., 2021), and multiplex sequencing of targeted fragments using an Illumina HiSeq. The library preparation protocol did not include a sonication step because gel electrophoresis of a subset of samples confirmed the presence of degraded DNA in extracts. DNA extracts were quantified via PicoGreen, and samples with total DNA below 10 ng were excluded from downstream sequencing, because initial tests indicated that captures were often successful in the 10–100 ng range. DNA input <10 ng may still be suitable in some cases; we did not thoroughly test samples below this threshold. Subsequent relatively standard library processing was performed by RAPiD Genomics, but notably all DNA extracts were cleaned with a standard SPRI bead protocol.

### Sequence Assembly

Raw reads were trimmed and quality filtered using Trimmomatic v. 0.39 (Bolger et al., 2014) to scan with a 20-base sliding window and cut when the average quality per base dropped below 15. We used FastQC (Andrews, 2010) to assess quality post-trimming. We measured sequencing success as the number of on-target reads divided by the total number of reads (“proportion-on-target reads recovered”). For each sample, the proportion-on-target reads recovered was calculated by mapping the trimmed reads to the 229 reference sequences used for bait design using BWA v. 0.7.17 (Li and Durbin, 2019) and processing results using samtools v. 1.10 (Li et al., 2009). Target loci were then assembled using a custom single-copy filtering pipeline based on Yang and Smith (2014) and aTRAM (Allen et al., 2018). After initial investigation, these locus assembly results were not included in our models due to the large impact of paralogy and other bioinformatic filtering criteria, potentially obscuring impacts due to specimen preservation alone.

### Data Analysis

Although we had sequence data from ∼15,000 samples, data cleaning and removal of samples lacking the desired predictors reduced our final dataset to 7,608 samples. All records with complete data for all predictor and response variables were used in a mixed modeling statistical framework. To evaluate the relationships between multiple predictors and the dependent variables of DNA yield and proportion-on-target reads recovered, we analyzed our dataset (Supplementary Table 1) using multivariate linear mixed regression models in the lme4 package (Bates et al., 2015) in R v. 4.0.2 (R Core Team, 2020). The predictors included in our models were (1) greenness, (2) specimen age, (3) herbarium, and (4) absolute latitude. We included plant families (all monophyletic and conformant to APG IV (2016) as a random effect based on the expectation that our predictor variables are related to our outcomes in a way that differs by plant family. Using family as a random effect serves as a proxy for species-specific traits such as secondary compounds, given that it is well known that certain plant groups are particularly problematic for DNA extraction (Adams et al., 1999; Neubig et al., 2014). We considered the interaction between certain predictors using models that included the interaction terms greenness ^∗^ specimen age as a predictor of percent-on-target-sequence and absolute latitude ^∗^ age as a predictor of DNA yield (Table 1).

**TABLE 1:** Formulas and summary statistics for all selected models included in this study.

Dependent variable	N	Fixed effects	Random effects	Marginal *R*^2^	Conditional *R*^2^	Adjusted *R*^2^	ΔAIC
DNA yield	7608	Absolute.Latitude * Specimen.Age + Greenness + Herbarium	Family	0.126	0.224	NA	16.04
Proportion-on-target reads recovered	7608	Greenness + Specimen.Age + Herbarium + Greenness * Specimen.Age	Family	0.112	0.716	NA	0.55
Proportion-on-target reads recovered	7608	DNA.Yield	None	NA	NA	0.00413118	29.49

For modeling the relationship between predictors and the dependent variable DNA yield, we used the R package arm (Gelman and Su, 2020) to standardize regression predictors of the models, and the step function in the R package lmerTest (Kuznetsova et al., 2017) for model selection based on automatic backward model selection of fixed and random parts of the linear mixed model. We checked our model terms for collinearity using the vif function in the R package car (Fox and Weisberg, 2019) and excluded any models with vif values over 5. Finally, we calculated an assessment of pseudo-*R*^2^ as one metric of model fit using the r.squaredGLMM function in the R package MuMIn (Barton, 2020). This assessment is based on Nakagawa and Schielzeth (2013) and provides a means to assess fit of just the fixed effects (*R*^2^_*m*_) and fixed and random efforts (*R*^2^_*c*_). We took a similar approach to model the relationship between predictors and the dependent variable proportion-on-target reads with modifications to account for the dependent variable being a proportion. In particular, we used the R package glmmTMB (Brooks et al., 2017) in order to utilize the beta error distribution appropriate for a proportional response variable, scaled predictors prior to modeling using the R package dplyr (Wickham et al., 2020), performed model selection using the dredge function in the R package MuMIn (Barton, 2020), checked for collinearity using the check_collinearity function in the R package performance (Lüdecke et al., 2020), and calculated pseudo-*R*^2^-values for the top model using the r2_nakagawa function in the R package performance (Lüdecke et al., 2020).

All analyses described above were performed on samples that passed initial quality checks, including a 10 ng cutoff for DNA yield. Because the question of whether any specimen is too old to sequence is of great interest, we performed a secondary analysis to determine whether omitting samples that did not meet DNA yield thresholds for sequencing biased our results related to specimen age. This allowed us to confirm that a failure to find a specimen age at which sequencing success drops off was not the result of a failure to sequence very old samples. For this analysis, we first randomly subsampled ∼300 of the samples that failed and a similar number of successes. After removing records for which specimen collecting dates were not available, we used a logistic regression to determine whether older specimens had higher probabilities of failing to meet the yield cutoff than younger ones.

## Results

### Assembled Dataset Characteristics

Our sampling included 23 families, but representation of families was uneven, reflective of differences in family species richness. About 60% of all specimens were from one family, Leguminosae (Fabaceae), 10% from Rosaceae, and seven other families (Urticaceae, Rhamnaceae, Moraceae, Polygalaceae, Fagaceae, Cucurbitaceae, and Begoniaceae) together comprised about 20% of all other specimens in approximately equal proportions. The remaining 14 families were represented by small numbers of specimens in our dataset. Out of a five-point scale, mean greenness score across all 7,608 samples was 2.31, corresponding to a score between “faded green” and “brown.” Twenty-six percent of the samples had a mean greenness score of 1–2 (“nice green” to “faded green”), 42% had a mean greenness score of > 2–3 (“faded green” to “brown”), and 31% had a mean score between > 3, and 4 (“brown” to “brown-black”); only 46 samples had a mean greenness score > four (“brown-black” to “black”; Supplementary Figure 1). Proportion-on-target reads recovered ranged from 0.02 to 0.99 with a mean of 0.34 and an interquartile range of 0.23. Specimen age ranged from 2 to 182 years with a mean of 39 years and an interquartile range of 24.

Total DNA yield across all specimens ranged from 0 to 4,449 ng with a mean of 994 ng and an interquartile range of 1,249. Although nearly all samples with DNA yields < 10 ng were excluded from sequencing and thus from this study, a small number of critical taxa with low DNA yields was sequenced. After selecting a subset of DNA extractions that were above and below our cutoff as described above, we ran a logistic regression and found that age of specimens did not explain odds of success or failure (i.e., the null and fitted models had nearly identical deviances). We also plotted the distribution of ages for the random samples above and below the cutoff in Supplementary Figure 2, which similarly shows that age does not impact success. We therefore conclude that excluding failed samples below our threshold from our analysis did not bias our results with regard to specimen age.

### Relationship Between DNA Yield and Specimen Characteristics

**FIGURE 2:**
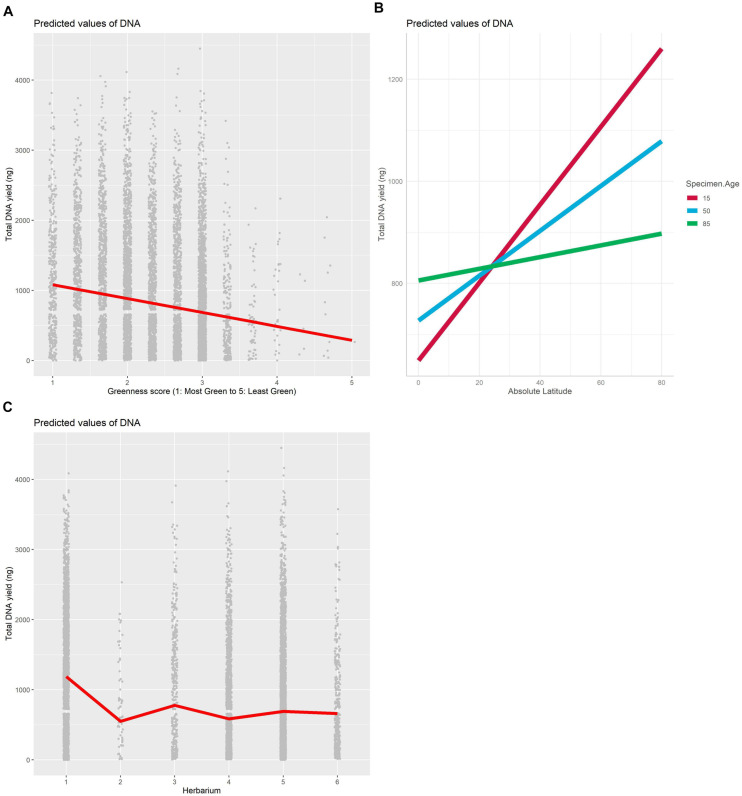
Plot of fixed effects “Greenness score” **(A)**, Herbarium **(B)** and “Absolute Latitude × Specimen Age” **(C)** included in the best mixed model with DNA as the dependent variable.

The best model for the relationship between DNA yield and specimen characteristics included the source herbarium, greenness, and the interaction between absolute latitude and age as fixed effects and family as a random effect (Table 1). The proportion of variation explained by fixed effects alone vs. fixed effects and the random effect indicates an important role for family in predicting DNA yield (marginal *R*^2^: 0.126, conditional *R*^2^: 0.224). Greenness was the most important specimen characteristic included in the selected model (Figure 2A and Supplementary Table 2), and herbarium 1 was clearly associated with higher DNA yield compared with the other five herbaria (Figure 2C and Supplementary Figure 2). We found that the interaction between specimen age and latitude had a significant but small effect (Supplementary Table 2), and that the relationship between absolute latitude and DNA yield was stronger for younger samples (Figure 2B).

### Relationship Between Targeted Sequencing Success and Specimen Characteristics

**FIGURE 3:**
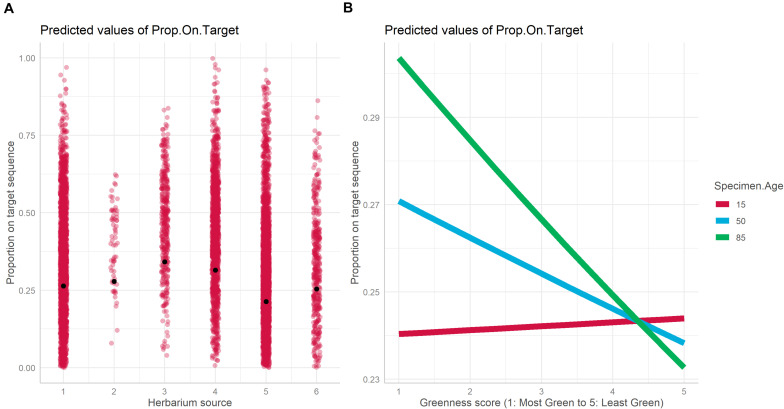
Plot of fixed effects “Herbarium” **(A)** and “Greenness score × Specimen Age” **(B)** included in the best mixed model with DNA as the dependent variable.

The best model for the relationship between proportion-on-target reads recovered based on specimen characteristics included specimen age, source herbarium, greenness, and the interaction between specimen age and greenness as fixed effects, and family as a random effect (Table 1). Absolute latitude was the only term dropped from the best model. The proportion of variance explained by fixed effects alone vs. fixed effects and the random effect indicates an important role for family in predicting proportion-on-target reads recovered (marginal *R*^2^: 0.112, conditional *R*^2^: 0.716). Herbarium was the fixed effect most important predictor of proportion-on-target reads recovered due to the strong association of herbarium 3 with higher proportion-on-target reads recovered compared with the other five herbaria (Figure 3A). We did not find significant interaction effects between herbarium and family, suggesting that taxonomic biases do not explain the importance of the herbarium predictor. Specimen age was also predictive of proportion-on-target reads recovered, and older age was associated with higher proportion-on-target reads recovered (Figure 3B). The interaction between specimen age and latitude was significant (Table 1). The relationship between greenness and proportion-on-target reads recovered was negative for younger samples (i.e., for younger samples, sample greenness was associated with lower proportion-on-target reads recovered) but became increasingly positive as sample age increased: for older samples, greener samples were associated with higher proportion-on-target reads recovered (Figure 3B).

### Relationship Between DNA Yield and Proportion-on-Target Reads Recovered

**FIGURE 4:**
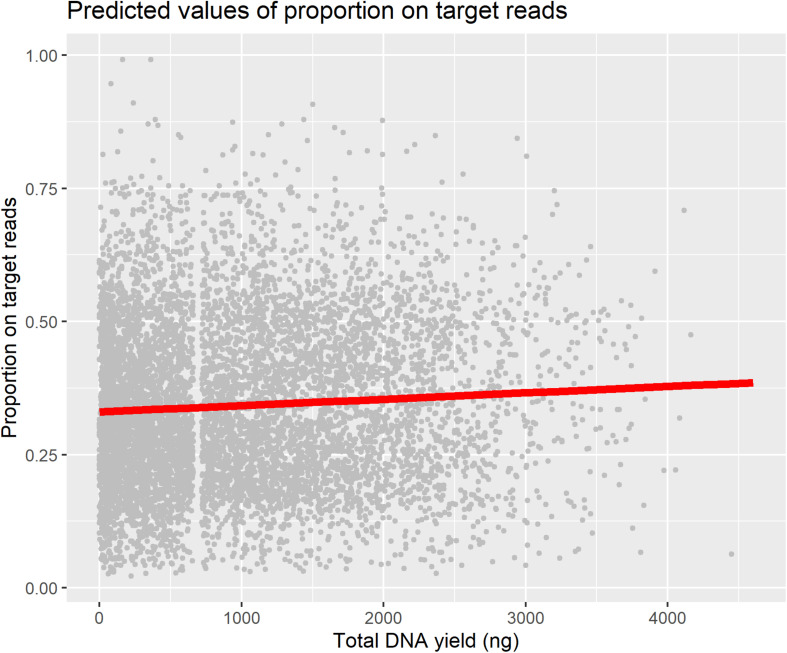
Plot of relationship between DNA yield and proportion-on-target-sequence with all data points plotted.

We used a linear mixed model to test for a predictive relationship between DNA yield and proportion-on-target reads recovered (Table 1), as this relationship is an important assumption in studies that use DNA yield as a proxy for sequencing success. We found only a weak positive relationship between DNA yield and proportion-on-target reads recovered, and plotting this relationship superimposed on all the data points illustrates the wide variation in proportion-on-target reads recovered independent of DNA yield (Figure 4).

## Discussion

Recent studies support the growing consensus that it is straightforward to obtain nuclear gene data from herbarium specimens using targeted-enrichment and short-read sequencing techniques (Hart et al., 2016; Kates et al., 2017; Vatanparast et al., 2018; Villaverde et al., 2018; Brewer et al., 2019; Couvreur et al., 2019; Gaynor et al., 2020; Stull et al., 2020), but which herbarium specimens are most suitable for this application has not been rigorously tested. Our sample size (nearly 8,000 herbarium specimens), specimen metadata collected (age, latitude, herbarium, greenness), and bait kit (designed to target 229 nuclear genes from across the rosids, comprising approximately a quarter of angiosperms) make ours by far the largest dataset available for investigating factors impacting targeted sequencing from herbarium specimens. The phylogenetic scope (the nitrogen-fixing clade, spanning ∼8% of angiosperms) and range of sample ages and quality make the results of this study broadly useful to all researchers in the development of herbarium specimen sampling strategies for targeted-sequencing projects.

### The Importance of Herbarium Source in Predicting DNA Yield and Targeted Sequencing Success

The herbarium source of a specimen was predictive of both DNA yield and proportion-on-target reads recovered, but the source herbaria did not predict these dependent variables in the same way (Figures 2C, 3A). Certain herbarium collections may disproportionately house samples from problematic families or tropical regions; however, low variance inflation factors and low values for correlation of fixed effects for all model terms (Supplementary Table 2) suggest that multicollinearity and correlation between herbarium and other effects such as latitude (cf. Neubig et al., 2014) are not responsible for the importance of herbarium in predicting DNA yield or proportion-on-target reads recovered. Herbarium-specific variables that could impact specimen DNA quality include archival mounting paper, ambient temperature and humidity, and differences in collector- or institution-specific sample preparation practices. It was not possible to include more about curatorial practices here because these are rarely reported; furthermore, we note that samples collected from all herbaria were successfully and informatively included in our phylogenetic dataset.

### Genomic DNA Yield and Targeted Sequencing Success

Most studies that consider how herbarium specimen characteristics are related to DNA sequencing evaluate the relationship between these characteristics and genomic DNA yield based on the assumption that DNA quality and yield are important factors that impact downstream targeted sequencing processes (e.g., Hart et al., 2016; Vatanparast et al., 2018; Villaverde et al., 2018; Brewer et al., 2019; Couvreur et al., 2019). In our study we do not find evidence that DNA yield is crucial to targeted sequencing success. However, due to the scale of our high-throughput sampling and sequencing approach, we used the Agilent 2100 Bioanalyzer (2100 Bioanalyzer Instrument (RRID:SCR_018043) and/or agarose gels to evaluate genomic DNA quality for only a small pilot subset of the samples in our study. Based on the molecular weight profiles of our pilot subsample, our methodology for library preparation was customized to exclude a fragmentation step. Therefore, it is likely that degraded DNA samples were more suitable for our sequencing strategy than non-degraded DNA.

### Specimen Age

Many studies have shown that herbarium storage over time contributes to DNA degradation (Sharma and Adams, 2010; Hart et al., 2016; Weiß et al., 2016). Our study included a broad age range from 2 to 182 years, with a mean age of 40 years. The effect of specimen age on DNA yield was not significant, and the specimen age term was dropped from the best model of the relationship between specimen characteristics and DNA yield. This result challenges previous interpretations of the importance of specimen age. However, not only was age the specimen characteristic most predictive of proportion-on-target reads recovered (a more directly useful metric for the design of targeted-enrichment projects), but this relationship was also positive: older sample age was associated with higher proportion-on-target reads recovered. This relationship clearly contradicts common practice of avoiding sampling of older specimens and strongly suggests researchers should not necessarily avoid specimens of any age.

The positive relationship between specimen age and higher proportion-on-target reads recovered is likely related to the fact that we omitted a DNA fragmentation step from our library preparation protocol after a review of the DNA molecular weight profile of our pilot sample set. Hart et al. (2016) found that no herbarium specimens more than 11 years contained high-molecular-weight DNA, so older specimens are likely better candidates for non-sonication library preparation. For throughput reasons, it was not possible to assess the size distribution of every single extraction, and some of the most recent specimens might have benefitted from moderate sonication while older specimens may have had more fragmented DNA present in the extracted DNA samples (Hart et al., 2016). The use of high-throughput molecular methods, with limited ability to tailor to individual samples, means our results can be taken as representative of large-scale phylogenomics projects. Considering the very low rate of sequencing failure and overall high proportion-on-target reads recovered across nearly all of our samples, we expect that when library preparation without fragmentation is used to process herbarium specimens, it is suitable to select older specimens to yield broad sequencing success. However, we did not sample many very old samples (Supplementary Table 1), so this recommendation may not apply to these specimens.

### Specimen Greenness

This is the first study of which we are aware to directly quantify specimen greenness as a predictor of DNA yield and sequencing success. Forrest et al. (2019) directly evaluated the impact of preservation techniques on targeted-sequencing by applying seven preparation methods to three fresh material accessions. However, directly evaluating the effects of these techniques broadly across taxonomic groups and over time is not possible because preservation histories are not recorded for most herbarium specimens (Brewer et al., 2019). Although many studies have shown that specimen preparation and preservation techniques can fragment DNA (Doyle and Dickson, 1987; Pyle and Adams, 1989; Staats et al., 2011; Särkinen et al., 2012), the relationship between these techniques and sequencing success has been unclear. Because greenness of specimen leaf tissue is thought to indirectly indicate the method of drying (Jankowiak et al., 2005), specimen greenness is a reasonable and quantifiable measure of specimen preservation; greener specimens were likely preserved using low heat (rather than no heat, high heat, or other preservatives such as ethanol dousing) and may therefore be expected to yield better quality DNA and sequencing results.

Among the specimen characteristics considered here, greenness was the most important predictor of DNA yield, but much less important for predicting proportion-on-target reads recovered. For both DNA yield and proportion-on-target reads recovered, specimens scored as greener had better results, but the effect was very weak for predicting proportion-on-target reads recovered. It is important to consider the interaction between specimen age and other predictors because older specimens collected before the present era of molecular biology were not collected with DNA preservation in mind and are more likely to have been dried under conditions that are poor for DNA preservation (Erkens et al., 2008). For younger specimens (considered here as those aged 0–20 years), we found a weak negative relationship between greenness and proportion-on-target reads recovered. However, with increasing specimen age, this relationship became stronger and increasingly positive (Figure 3B), suggesting that preferentially selecting greener specimens may be more important when sampling from older specimens. The same interaction was not seen when considering the effect of this interaction on DNA yield (data not shown).

An additional concern related to the effect of specimen preservation on DNA yield and sequencing success is that samples collected in forested tropical regions are more likely to have been treated with alcohol immediately after collection (the “Schweinfurth method”; Schrenk, 1888) to prevent fungal and bacterial growth that may occur under high humidity prior to drying (Forrest et al., 2019). As mentioned above, it is not always clear whether a specimen has been treated in this way. Although more recent tropical collections are often annotated with the method of preservation on specimen labels, very few specimens included in our study were annotated in this way. The absolute latitude predictor included in our models may serve as a proxy, as specimens collected closest to the Equator are more likely to have been preserved using treatments that may degrade DNA (Forrest et al., 2019), and collection in drier, colder conditions in more temperate and polar regions may be more likely to limit immediate DNA degradation.

Absolute latitude was strongly positively associated with higher DNA yield (that is, samples from higher latitudes yielded more DNA) but was not a significant predictor of proportion-on-target reads recovered. As described above, our no-fragmentation library building protocol likely minimizes or eliminates the expected negative effect of degraded DNA on sequencing success; if specimens collected closer to the Equator are more likely to have degraded DNA, this may affect DNA extraction yield but not sequencing success. Because distance from the Equator may be more important for older specimens as collecting practices that damage specimens have become less common in recent years, we considered the effect of the interaction between absolute latitude and specimen age. The strength of the relationship between distance from the Equator and DNA yield decreased with increasing specimen age (Figure 2B), suggesting that older specimens from tropical regions are no more likely to yield low amounts of DNA than more recently collected specimens from tropical regions despite the difference in preservation practices over time. However, for a given genus in our target taxon list, our collecting teams always sampled the specimens that appeared to be of the highest quality available, so even without preservation methods annotated, it is possible that we avoided sampling many older specimens preserved using DNA-degrading methods.

### Taxonomic Group

**FIGURE 5:**
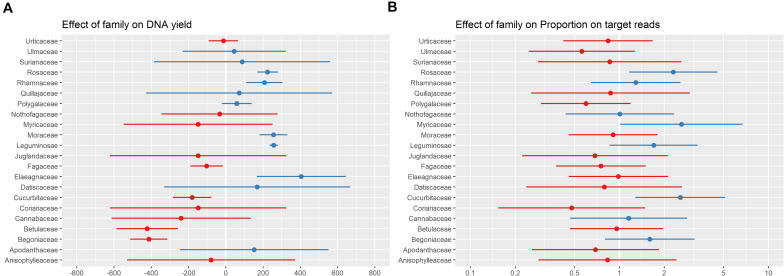
Plot of random effect (plant family) for the best models of the relationship between specimen characteristics and DNA yield **(A)** and proportion-on-target-sequence **(B)**. The range and mean are shown in blue for families with a higher values than the overall mean DNA yield and proportion-on-target-sequence, and red for families with lower values.

Taxonomic group (considered by including family sensu APG IV (2016) as a random effect in all models) was more predictive of both DNA yield and proportion-on-target reads recovered than any other factor included in the models (Figure 5). A previous assessment of the effect of patristic distance between sample and taxa included in the bait design on sequencing success showed only a very weak relationship between the two (Folk et al., 2021), suggesting that the variation in proportion-on-target reads recovered was not due to how closely related a sample was to the taxa included in bait design. Instead, this variation is likely related to the traits specific to a taxonomic group (e.g., leaf texture or presence of secondary compounds) that influence both DNA extraction and targeted-enrichment protocols. We did not adjust our protocol for individual taxonomic groups due to our sampling and wet-lab workflows in which samples were not organized by taxonomic groups prior to DNA extraction or sample submission for sequencing. Some of the families associated with a negative effect on DNA yield are known to be problematic, and resources are available for improving DNA extractions for these families (e.g., Begoniaceae: Kopperud and Einset, 1995; Cucurbitaceae: Brown et al., 1998; Urticaceae: Sarrazola and Alzate, 2019). There are many possible adjustments to DNA extraction protocols to address issues that may improve DNA products (e.g., high salt and sorbitol pre-soaking: Inglis et al., 2018; down-stream PCR enhancers: Samarakoon et al., 2013; use of commercial solutions: Abdel-Latif and Osman, 2017; STE and HEPES for polysaccharide-rich tissue: Shepherd and McLay, 2011). However, it remains unclear how to modify DNA extraction protocols to increase sequencing success; we have shown here that DNA yield is not strongly predictive of sequencing success (Figure 4 and Table 1), and those families associated with high DNA yield differed from those associated with high proportion-on-target reads recovered (Figure 5).

In sum, we found that specimen characteristics (i.e., specimen age, greenness, and collection latitude) only explain a modest proportion of the variation in DNA yield or proportion-on-target reads recovered, and that family, which is harder to control and treated here as a random effect, was more important (Table 1). Among the fixed effects, herbarium, which was also the most difficult to control, was most predictive of DNA yield and sequencing success (Supplementary Table 2). These results suggest that for projects that massively sample across wide phylogenetic breadth it may not be worth prioritizing specimen quality factors over adjustments to broader project design that could promote sequencing success; however, this is rarely a tradeoff that can be considered because selection of plant families and herbarium collections are determined by project goals and sampling efficiencies.

## Conclusion and Recommendations

A key goal of this study was to guide best practices for collecting and sequencing herbarium specimens for targeted sequencing projects. Broad inclusion of herbarium specimens in phylogenomic studies is potentially the most important remaining frontier for generating the comprehensive global phylogenies needed to answer the most compelling long-standing questions in plant ecology and evolution.

Using a sample set of nearly 8,000 herbarium specimens, we have demonstrated that when DNA extraction and library-building protocols are designed with herbarium specimens in mind, most herbarium specimens will provide suitable sequence for phylogenetic datasets. Many factors thought to be problematic for DNA extraction in this and other studies are not unilaterally associated with poor sequencing outcomes. When fragmentation was not included in library preparation, older specimens had higher proportion-on-target reads recovered than younger specimens, but most specimens had suitably high proportions-on-target-sequence for standard phylogenomic applications. This finding stands in contrast to typical advice for destructive sampling of museum specimens, which stresses the avoidance of old specimens. Indeed, some herbaria have explicit policies against sampling specimens beyond a certain age, often due to perceived success with these materials as well as historical value. On the whole, many factors more closely related to sequencing success, such as phylogenetic placement, are difficult or impossible to optimize in practice.

Based on our findings, we make the following recommendations:

1. DNA extraction and library-building protocols should be established and tested prior to broad-scale sampling of herbarium specimens, as it is likely that our exclusion of sonication is responsible for the favorable performance of old specimens.

2. DNA yield is not an important predictor of sequencing success for Hyb-Seq approaches. Thus, researchers can in most cases skip fine-tuning DNA extraction protocols to optimize yield. Researchers should also not preferentially sample herbarium specimens that they believe are likely to yield high amounts of genomic DNA at the expense of other considerations more related to sequencing success.

3. Researchers should not necessarily avoid sampling old specimens from tropical climates, as these samples have good sequencing success where suitable library-building protocols are used.

4. Taxon identity, probably indirectly representing taxon-specific traits such as secondary compound content, affects sequencing success more than any other predictor, and potential taxon-specific extraction issues should be identified early on, so that DNA extraction protocols can be optimized for certain taxonomic groups to ensure downstream sequencing success. This is likely not sufficiently important to require complete protocol separation of samples by taxonomic group, but care is recommended for any highly sampled taxonomic groups thought to have secondary compounds or other characteristics that may affect sequencing success.

## Data Availability Statement

The original contributions presented in the study are included in the article/Supplementary Material, further inquiries can be directed to the corresponding author/s.

## Author Contributions

JD, RG, and RF conceived of the study. RG, RF, HK, PS, and DS collected herbarium specimens for the Nitfix project that were used in this study. HK extracted DNA, assembled sequence data, and curated sequence metadata that were used to assemble this dataset. CS assisted with sequence metadata curation. JD and RG designed the greenness scoring project and JD trained volunteers who assisted in compiling greenness scores. RL built and managed specimen metadata databases, including managing all images and interfacing with Notes from Nature for label data generation. RG developed the analytical framework and HK performed the statistical analysis. All authors contributed to the final manuscript.

## Conflict of Interest

The authors declare that the research was conducted in the absence of any commercial or financial relationships that could be construed as a potential conflict of interest.

## Supplementary Material

Supplementary Figure 1 Distribution of greenness scores across 7,974 herbarium specimens with 1 being the most green to 5 being the least green.

Supplementary Figure 2 Comparison of the spread of specimen ages between 300 random subsamples of specimens that did and did not pass the DNA cutoff (10 ng) for sequencing.

Supplementary Table 1 Specimen metadata that comprise the dataset used for all models in this study.

Supplementary Table 2 Model summaries and variance inflation coefficients for the two mixed models.
