# Supplementary figures and images for: The Effects of Herbarium Specimen Characteristics on Short-Read NGS Sequencing Success in Nearly 8000 Specimens: Old, Degraded Samples Have Lower DNA Yields but Consistent Sequencing Success
Source: Front Plant Sci. 2021 Jun 23;12:669064. doi: 10.3389/fpls.2021.669064 (PMC8262526; doi:10.3389/fpls.2021.669064)

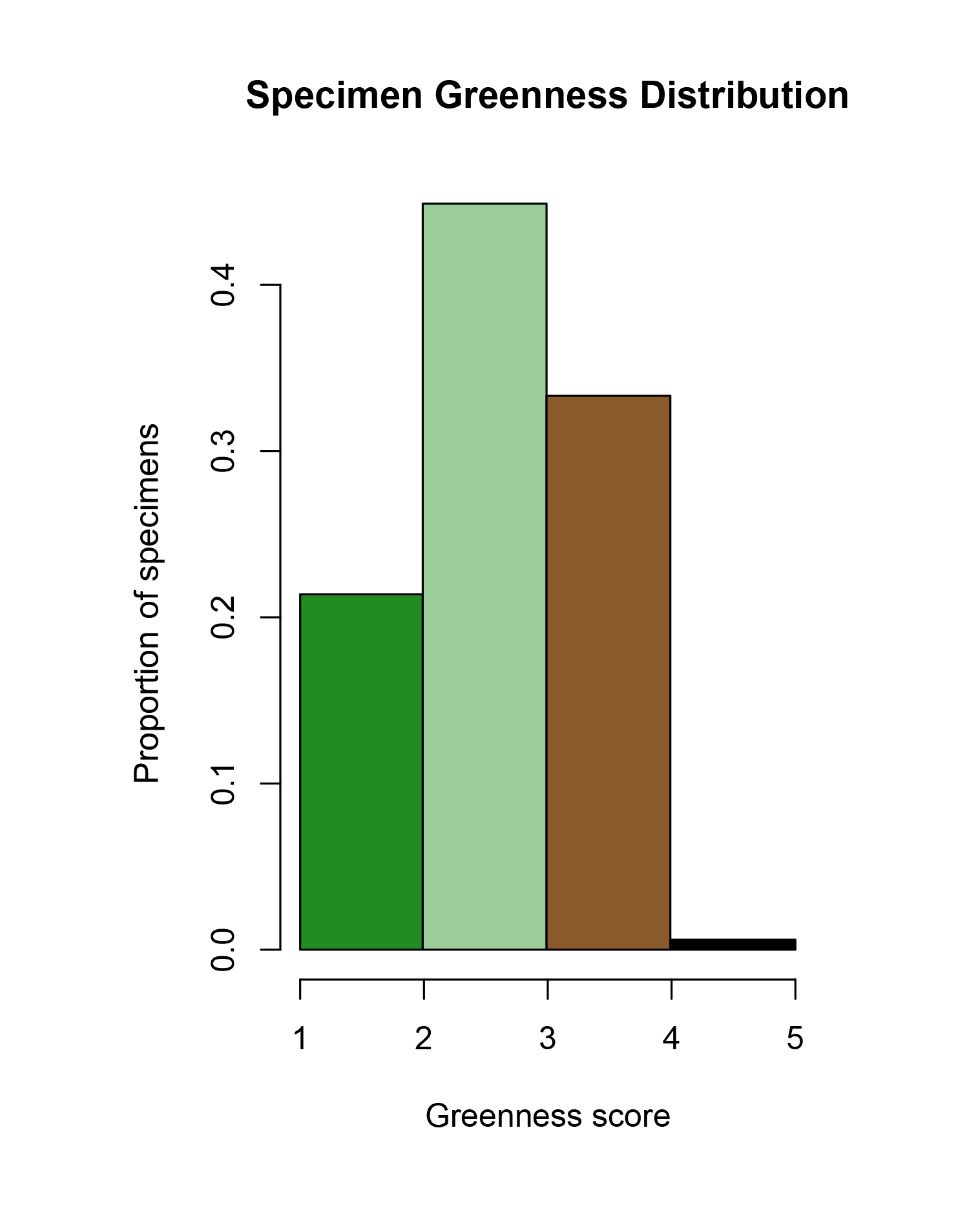

Supplement: Supplementary Figure 1 — Distribution of greenness scores across 7,974 herbarium specimens with 1 being the most green to 5 being the least green. [file Image_1.TIF]

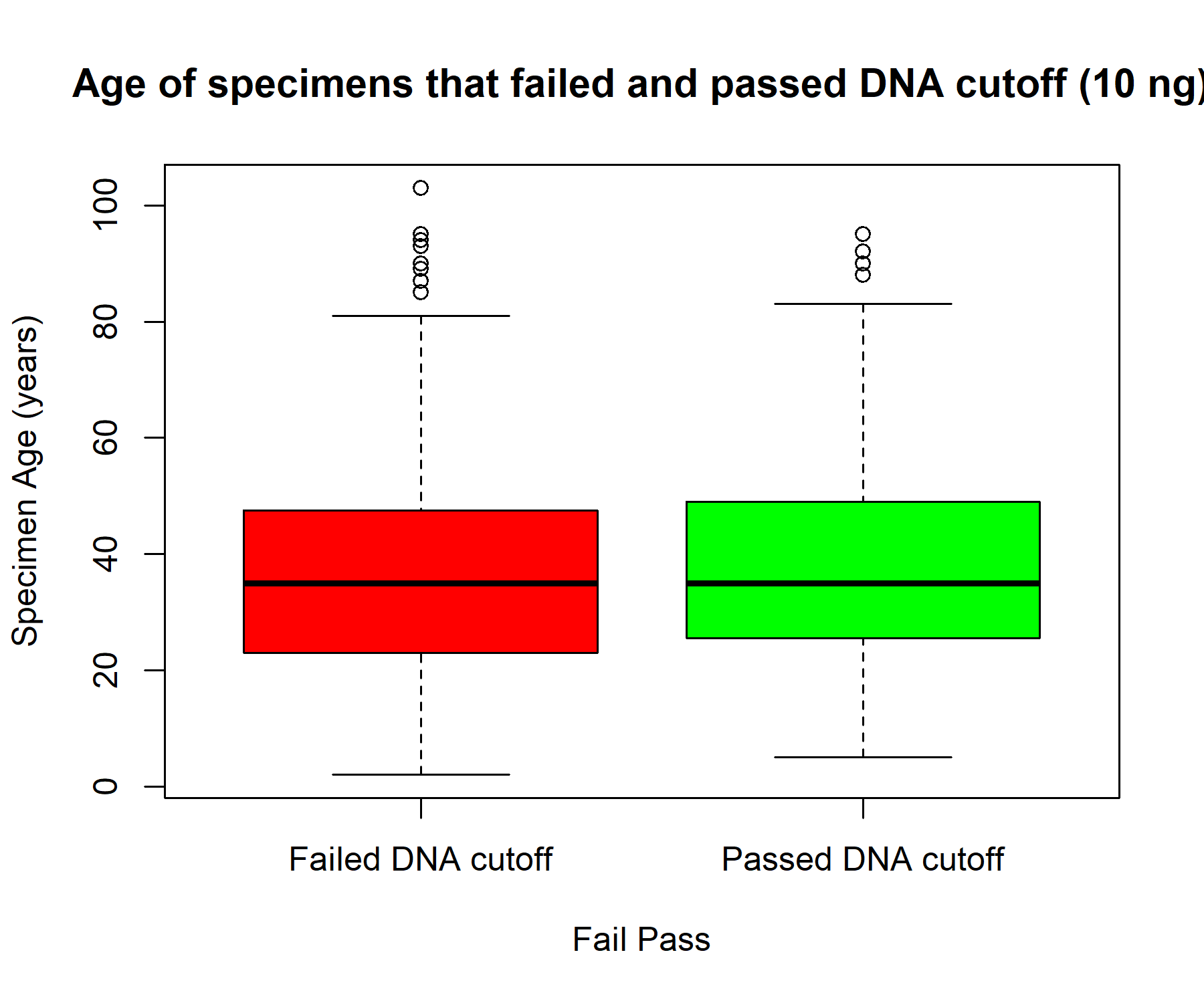

Supplement: Supplementary Figure 2 — Comparison of the spread of specimen ages between 300 random subsamples of specimens that did and did not pass the DNA cutoff (10 ng) for sequencing. [file Image_2.TIFF]
